# Is the Sense of Coherence Associated With Flossing Among Southern Brazilian Male Adolescents? A Cross‐Sectional Study

**DOI:** 10.1111/idh.70018

**Published:** 2025-12-02

**Authors:** Nicássia Cioquetta Lock, Maria Laura Castro Alves Ribeiro Gazola, Patrícia Kolling Marquezan, Julio Eduardo do Amaral Zenkner, Luana Severo Alves

**Affiliations:** ^1^ Department of Restorative Dentistry Federal University of Santa Maria Santa Maria Brazil; ^2^ Department of Microbiology and Parasitology Federal University of Santa Maria Santa Maria Brazil; ^3^ Department of Stomatology Federal University of Santa Maria Santa Maria Brazil

**Keywords:** adolescent, cross‐sectional study, flossing, oral hygiene, sense of coherence

## Abstract

**Objective:**

To assess the association between sense of coherence (SoC) and flossing among southern Brazilian male adolescents aged 18–19 years.

**Background:**

A high SoC has been reported to be associated with beneficial oral health‐related habits and behaviours but no study has properly assessed its association with flossing.

**Methods:**

A cross‐sectional study was conducted and included 18–19‐year‐old adolescents who joined the Brazilian Army as enlisted for mandatory military service in two military bases located in southern Brazil (*n* = 507). A structured questionnaire was applied to gather data on flossing (use and frequency) and SoC, which was assessed using the validated Brazilian short version of the SoC scale (SOC‐13). The outcomes of this study were flossing, regardless of frequency, and daily flossing, both of which were defined as binary variables (‘no’ or ‘yes’). The main predictor variable was SoC, categorised as low, moderate or high. The association between SoC and flossing was investigated using Poisson regression models. Unadjusted and adjusted prevalence ratios (PR) and their respective 95% confidence intervals (CI) were estimated.

**Results:**

The prevalence of flossing, regardless of frequency and daily flossing was 34.4% and 13%, respectively. Adolescents with high SoC were 43% more likely to floss than those with low SoC (adjusted PR = 1.43; 95% CI = 1.06–1.93). Similarly, adolescents with high SoC had around two‐fold greater likelihood of daily flossing than their counterparts with low SoC (adjusted PR = 2.18; 95% CI = 1.20–3.96).

**Conclusion:**

This study showed a significant association between high SoC and a greater likelihood of flossing among 18–19‐year‐old male adolescents from southern Brazil.

## 1 Introduction

Sense of coherence (SoC) is a psychological resource related to a person's ability to cope with environmental stress and life tensions [1]. It comes from the salutogenic theory and is defined as a global orientation of the person through which life is understood as more or less comprehensible, meaningful and manageable. A person with a higher SoC is able to find suitable solutions when facing challenges in his/her life [2].

SoC has been reported to be associated with oral health‐related habits and behaviours, such as tooth brushing frequency, dental attendance pattern and dietary habits. Three systematic reviews on this topic showed that individuals with higher SoC were more likely to (i) report a higher frequency of tooth brushing and (ii) visit a dentist preventively, and less likely to eat sugary food/drinks between meals [3, 4, 5]. To the best of our knowledge, only one previous study investigated SoC as a potential predictor of flossing [6]. Although the authors found a higher mean SoC score among individuals who used to perform interproximal cleaning regularly, this variable was not included in the risk models. In addition, this study combined two different methods of interproximal cleaning (dental floss and toothpicks).

The benefits of flossing for the control of interproximal caries have been questioned since the early 2000's [7] and we still lack evidence on this topic; however, flossing in addition to tooth brushing may reduce gingivitis or plaque, or both, more than tooth brushing alone [8]. In this sense, the Brazilian Ministry of Health recommends the use of dental floss as a complement to daily brushing to maintain oral health [9] and the American Dental Association (ADA) recommends flossing at least once a day for proximal cleaning [10]. Despite these recommendations, convincing individuals to use dental floss is challenging. Children and adolescents generally do not engage in this practice, and a lack of motivation has been described as one of the reasons [11]. In this context, the investigation of a psychological resource that could influence oral hygiene habits such as SoC might be of great value. Therefore, the aim of the present study was to assess the association between SoC and flossing among southern Brazilian male adolescents aged 18–19 years. We hypothesised that a higher SoC affords a greater likelihood of flossing.

## 2 Methods

This cross‐sectional study was conducted in two Brazilian Army military bases located in southern Brazil and included all adolescents aged 18–19 years who were enlisted for mandatory military service.

Data collection was carried out in 2019–2020 in the city of Itaqui, RS (estimated population around 37,000 inhabitants), and in 2021 in the city of Santiago, RS (estimated population around 50,000 inhabitants), and included the application of two questionnaires. A structured questionnaire gathered data on socio‐demographic information and oral health‐related habits, such as education level, family income, dental flossing and whether the participant had visited a dentist for preventive purposes in the previous year. To assess the study outcomes, two questions were applied. The first one was ‘Do you use dental floss?’, and the answer alternatives were ‘no’ or ‘yes’, with no mention of flossing frequency. For those who answered ‘yes’, a second question was then applied: ‘How often do you floss?’, and the answer alternatives were ‘sometimes’, ‘once a week’, ‘once every two days’, or ‘once a day’.

SoC was assessed using the validated Brazilian short version of the SoC scale (SOC‐13) [12], which is composed of 13 items divided into three components: comprehensibility, manageability, and meaningfulness. Response options are presented on a five‐point Likert scale, in which the sum of points can range from 13 to 65. The higher the SoC score, the higher the person's ability to cope with stressful situations.

### 2.1 Data Analysis

The outcomes of this study were flossing, regardless of frequency, and daily flossing, both of which were defined as a binary variable (‘no’ or ‘yes’). For the definition of the outcome daily flossing, categories ‘sometimes’, ‘once a week’ and ‘once every two days’ were grouped as ‘no’ and ‘once a day’ was considered ‘yes’. The main predictor variable was SoC, categorised as low, moderate, or high, based on tertiles, as previously described in the literature [13]. Other predictors included in the study as adjusting variables were education level (categorised as < 8 years, 8–10 years, or ≥ 11 years), family income (≤ 3 Brazilian minimum wages (BMW, being 1 BMW corresponding to approximately 200 US dollars during the period of data collection) or ≥ 4 BMW) and dental visit in the last year for preventive purposes (dichotomized as ‘no’ or ‘yes’).

The Wald test was preliminarily used to compare the prevalence of flossing and daily flossing between categories of explanatory variables. The association between SoC and the study outcomes was investigated using Poisson regression models. Unadjusted and adjusted prevalence ratios (PR) and their respective 95% confidence intervals (CI) were estimated. Variables with *p* < 0.20 in the unadjusted analysis were included in the adjusted model. Data analysis was performed using STATA software (Stata 14.2, Stata Corporation, College Station, USA) and the level of significance was set at 5%.

### 2.2 Ethical approval

The study protocol was approved by the Research Ethics Committee of the Federal University of Santa Maria (CAAE 20079519.1.0000.5346). The research was conducted ethically, in accordance with the World Medical Association Declaration of Helsinki. All participants provided written informed consent.

## 3 Results

All invited individuals agreed to participate. A total of 520 adolescents were examined; however, this study includes data on 507 individuals because 13 had missing information on the SoC scale. The prevalence of flossing was 34.4%, corresponding to 173 individuals. Regarding daily flossing, this prevalence was 13.0% (*n* = 66 adolescents). Sample distribution and the prevalence of flossing and daily flossing according to explanatory variables are presented in Table 1. The prevalence of flossing was significantly higher in adolescents with high SoC (41.2%) than in those with low SoC (29.2%). Similar results were found for daily flossing, with a prevalence of 18.6% among adolescents with high SoC and 8.7% among those with low SoC (*p* < 0.05). Adolescents whose families earned ≥ 4 BMW had a higher prevalence of flossing than those whose families earned ≤ 3 BMW, which was consistently found for both study outcomes.

**TABLE 1:** Sample distribution and the prevalence of flossing and daily flossing according to explanatory variables.

	*n* (%)	Flossing	Daily flossing
		% (95% CI)	% (95% CI)
Education level			
< 8 years	75 (14.8)	30.1 (19.5–40.8)^a^	6.8 (1.0–12.5)^a^
8–10 years	182 (35.9)	30.8 (24.0–37.5)^a^	11.0 (6.4–15.5)^ab^
≥ 11 years	250 (49.3)	38.3 (32.2–44.4)^a^	16.4 (11.8–21.0)^b^
Family income			
≤ 3 BMW	380 (74.9)	30.0 (25.4–34.7)^a^	10.8 (7.7–14.0)^a^
≥ 4 BMW	127 (25.1)	47.2 (38.5–56.0)^b^	19.7 (12.7–26.6)^b^
Sense of coherence			
Low	161 (31.8)	29.2 (22.1–36.2)^a^	8.7 (4.3–13.1)^a^
Moderate	178 (35.1)	32.8 (25.8–39.7)^ab^	11.8 (7.0–16.6)^ab^
High	168 (33.1)	41.2 (33.7–48.8)^b^	18.6 (12.6–24.5)^b^
Dental visit in the last year for prevention			
No	412 (81.4)	31.5 (27.0–36.1)^a^	11.7 (8.6–14.8)^a^
Yes	94 (18.6)	47.3 (37.1–57.5)^b^	19.1 (11.1–27.2)^a^
Total	507 (100)	34.4 (30.2–38.6)	13.0 (10.0–16.0)

*Note:* Different letters indicate statistically significant difference between categories (*p* < 0.05, Wald test).

Abbreviations: BMW, Brazilian minimum wage; CI, confidence interval.

Table 2 shows the association between explanatory variables and flossing, regardless of frequency. All variables were significantly associated with dental flossing in the unadjusted and adjusted models, with the exception of education level. After adjusting for important cofactors, adolescents with high SoC were 43% more likely to floss than those with low SoC (PR = 1.43; 95% CI = 1.06–1.93). Individuals with higher family income (PR = 1.52; 95% CI = 1.19–1.92) and those who visited a dentist for prevention (PR = 1.50; 95% CI = 1.16–1.94) were about 50% more likely to floss.

**TABLE 2:** Association between explanatory variables and flossing, regardless of frequency (unadjusted and adjusted Poisson regression models).

	Unadjusted			Adjusted		
	PR	95% CI	*p*	PR	95% CI	*p*
Education level						
< 8 years	1.00					
8–10 years	1.02	0.68–1.54	0.92			
≥ 11 years	1.27	0.87–1.87	0.22			
Family income						
≤ 3 BMW	1.00			1.00		
≥ 4 BMW	1.57	1.24–2.00	< 0.001	1.52	1.19–1.92	0.001
Sense of coherence						
Low	1.00			1.00		
Moderate	1.12	0.81–1.55	0.48	1.15	0.84–1.58	0.39
High	1.41	1.04–1.91	0.02	1.43	1.06–1.93	0.02
Dental visit in the last year for prevention						
No	1.00			1.00		
Yes	1.50	1.16–1.94	0.002	1.50	1.16–1.94	0.002

Abbreviations: BMW, Brazilian minimum wage; CI, confidence interval; PR, prevalence ratio.

As for daily flossing, adolescents with high SoC had around two‐fold greater likelihood of flossing than their counterparts with low SoC (PR = 2.18; 95% CI = 1.20–3.96), even after the adjustment for other cofactors (Table 3). Visiting a dentist in the last year for prevention also remained significantly associated with daily flossing in the adjusted model (PR = 1.69; 95% CI = 1.05–2.74).

**TABLE 3:** Association between explanatory variables and daily flossing (unadjusted and adjusted Poisson regression models).

	Unadjusted			Adjusted		
	PR	95% CI	*p*	PR	95% CI	*p*
Education level						
< 8 years	1.00			1.00		
8–10 years	1.63	0.63–4.18	0.31	1.51	0.59–3.81	0.39
≥ 11 years	2.43	0.99–5.92	0.05	2.11	0.86–5.15	0.10
Family income						
≤ 3 BMW	1.00			1.00		
≥ 4 BMW	1.82	1.15–2.87	0.01	1.56	0.99–2.46	0.054
Sense of coherence						
Low	1.00			1.00		
Moderate	1.36	0.71–2.58	0.35	1.42	0.75–2.68	0.28
High	2.13	1.18–3.86	0.01	2.18	1.20–3.96	0.01
Dental visit in the last year for prevention						
No	1.00			1.00		
Yes	1.64	1.00–2.69	0.05	1.69	1.05–2.74	0.03

Abbreviations: BMW, Brazilian minimum wage; CI, confidence interval; PR, prevalence ratio.

The calculation of the study power was performed for the prevalence of flossing and daily flossing between non‐exposed (low SoC) and exposed (high SoC) individuals using 95% CI and values of 63% and 74% were observed, respectively.

## 4 Discussion

This study investigated the association between SoC and flossing among southern Brazilian male adolescents aged 18–19 years. Findings from the present study demonstrated that adolescents with high SoC were more likely to floss than those with low SoC, even after the adjustment for important cofactors. To the best of our knowledge, this is the first study addressing this issue.

Flossing has been recommended as an adjunct to tooth brushing to maintain oral health; however, patients' compliance with flossing is not as great as with tooth brushing. It is possible to speculate that factors such as the longer time spent on oral hygiene procedures, the need for greater manual dexterity, and the additional cost may explain, at least in part, this fact. A previous study showed that low compliance and difficulties in flossing among children and adolescents seemed to be more related to lack of motivation, although problems concerning manual skills were also observed [11]. Previous studies have investigated the factors associated with the habit of flossing, such as socioeconomic and environmental factors [14, 15, 16]. Moraes et al. [14] found that children from families with higher income and maternal schooling presented a higher prevalence of dental floss use. Soofi et al. [15] found that flossing was significantly associated with high socioeconomic status. They showed that socioeconomic status and education level made the most positive contributions to socioeconomic inequality in flossing behaviour. The study by Mata et al. [16] evaluated the socioeconomic inequalities in oral health‐related behaviours, such as frequency of tooth brushing, flossing, and dental appointments, in 18‐year‐old Portuguese adolescents. The authors showed that socioeconomic inequalities in health‐related behaviours were associated with employment status and educational level, adolescent educational level, sex, and residential area. These findings were corroborated by the present study, since a higher family income was significantly associated with flossing. When it comes to daily flossing, a borderline *p*‐value for this association was detected (*p* = 0.054), probably due to the reduced number of individuals who reported daily flossing (*n* = 66), thus reducing the statistical power for this outcome.

The literature is scarce regarding studies addressing the association between SoC and the habit of flossing. After revising the existing literature extensively, we could find six studies including SoC and flossing. In the studies by Peker et al. [17] carried out in Turkey and Reddy et al. [18] in India, SoC was modelled as the outcome, not the predictor. In three other studies, SoC and flossing were both included as possible predictors of periodontal outcomes, dental caries, and dental pain [19, 20, 21]. The only study investigating SoC as a possible predictor of flossing was conducted by Lindmark et al. [6] and included 910 Swedish individuals aged 20–80 years. The authors presented only a preliminary analysis between SoC and flossing by comparing the mean (±standard deviation) SoC scores between individuals who performed interproximal cleaning regularly (71.3 ± 10.6) and those who did not (69.1 ± 11.9), and a significant difference was found (*p* = 0.03). Despite this finding, the variable was not included in the adjusted models. Furthermore, as stated previously, this study combined flossing with other methods of interproximal cleaning (toothpick). The present study showed that adolescents with high SoC were 43% more likely to floss than those with low SoC. Similarly, daily flossing among adolescents with high SoC was twice as likely as among those with low SoC. Considering that flossing presumes sufficient motivation of the individual, a high SoC may make the flossing effort seem worthwhile for these adolescents. This finding is coherent with the significant association between SoC and the habit of brushing teeth ≥ 3 times/day observed in this sample of adolescents (data not shown). Considering these aspects, strategies to improve SoC during adolescence might ultimately enhance oral hygiene habits and oral health. Although interventions to improve SoC in school‐based oral health promotion settings have shown promising results [22, 23], no study has investigated this issue in the age group under investigation in the present study. Further intervention studies may elucidate whether addressing SoC is a feasible strategy to be adopted in oral health promotion programmes targeting older adolescents or even young adults.

Limitations of our study include its cross‐sectional design, which prevents us from defining causal inferences, and its convenience sample that cannot be considered representative of the whole population of 18–19‐year‐old adolescents from southern Brazil. All recruits joining the military service were included in the study by convenience and no sample size calculation was performed a priori. Although the statistical power did not reach the usually adopted threshold of 80%, the sample of 507 adolescents was considered of sufficient size to detect a significant association between SoC and flossing. Considering that interdental cleaning with interdental brushes (IDBs) is the most effective method for interdental plaque removal [24], it could be argued that other devices could have been investigated in the present study. We have, in fact, asked whether the participants used other interdental cleaning devices and 25% (*n* = 130) reported using woodsticks. None of them reported using IDBs, probably due to the lack of open interdental spaces in this age group. Some strengths of the present study also have to be pointed out. The sample was composed of a homogeneous population in terms of sex and age, which seems to be an advantage, since it eliminates the possible influence of these factors on the study findings. Finally, the study was conducted during mandatory military service, thus including individuals who probably were involved with physically and psychologically stressful situations, thus making this a potentially interesting scenario for studying psychological resources, such as SoC.

## 5 Conclusion

This cross‐sectional study showed a significant association between high SoC and a greater likelihood of flossing among 18–19‐year‐old male adolescents from southern Brazil.

## 6 Clinical Relevance

### 6.1 Scientific Rationale for Study

Sense of coherence (SoC) is a psychological resource related to a person's ability to cope with environmental stress and life tensions. Although SoC has been reported to be associated with oral health‐related habits and behaviours, no previous study has investigated its association with flossing.

### 6.2 Principal Findings

A significant association between SoC and dental flossing among 18–19‐year‐old male adolescents from southern Brazil was found; the higher the SoC, the greater the likelihood of flossing.

### 6.3 Practical Implications

Strengthening the SoC may promote better oral hygiene practices among adolescents, thus contributing to controlling oral diseases.

## Author Contributions

N.C.L. collected the data and wrote the manuscript; M.L.C.A.R.G. helped the main researcher during data collection, prepared the dataset for statistical analysis, and wrote the manuscript; P.K.M. calibrated the main researcher and wrote the manuscript; J.E.A.Z. and L.S.A. analysed the data and wrote the manuscript. All authors reviewed the manuscript and gave their final approval.

## Funding

We acknowledge the support of the National Coordination of Post‐graduate Education (‘Coordenação de Aperfeiçoamento de Pessoal de Nível Superior’—CAPES), Ministry of Education, Brazil (funding code 001) and the Federal University of Santa Maria (‘Universidade Federal de Santa Maria’).

## Conflicts of Interest

The authors declare no conflicts of interest.

## Data Availability

The data that support the findings of this study are available from the corresponding author upon reasonable request.
